# Nerve Combing for Idiopathic Trigeminal Neuralgia Without Neurovascular Compression

**DOI:** 10.4317/jced.63726

**Published:** 2026-02-26

**Authors:** Yoshihito Maruyama, Keita Takizawa, Kana Kawai-Ozasa, Andrew Young, Naoki Otani, Noboru Noma

**Affiliations:** 1 DDS, Department of Oral Medicine, Nihon University school of Dentistry Tokyo, Japan; 2 DDS, MSD Department of Diagnostic Sciences, Arthur Dugoni School of Dentistry, University of the Pacific, San Francisco, United States; 3 MD, Department of Neurological Surgery, Nihon University School of Medicine, Tokyo, Japan, 1-6 Kandasurugadai, Chiyoda-ku, Tokyo 101-8309, Japan

## Abstract

Trigeminal neuralgia (TN) is classified into classical, secondary, and idiopathic forms, but the pathophysiology and optimal surgical management of idiopathic TN remain controversial, especially in the absence of definite neurovascular compression. We report two cases of idiopathic TN successfully treated with nerve combing combined with posterior fossa exploration. A 44-year-old woman and a 73-year-old man presented with medically refractory paroxysmal facial pain, and preoperative MRI demonstrated vascular contact without clear compression. Intraoperatively, thickened arachnoid adhesions and axial torsion of the trigeminal nerve root were identified. Meticulous arachnoid dissection restored normal nerve alignment, followed by nerve combing. In one case, intraoperative compound nerve action potential monitoring suggested reduced neural hyperexcitability. Both patients achieved complete postoperative pain relief with only transient sensory disturbances. These cases indicate that axial torsion of the trigeminal nerve root caused by arachnoid adhesions may play a role in the pathophysiology of idiopathic TN. A diagnostic trial of carbamazepine can serve as a valuable adjunct for the clinical diagnosis of orofacial pain disorders suggestive of trigeminal neuralgia, even in the absence of neurovascular compression on MRI. Furthermore, nerve combing combined with meticulous arachnoid dissection may represent an effective surgical strategy for select patients.

## Introduction

According to the International Classification of Orofacial Pain, 1st edition (ICOP-1), trigeminal neuralgia is categorized into classical trigeminal neuralgia, secondary trigeminal neuralgia, and idiopathic trigeminal neuralgia (1). Classical trigeminal neuralgia is diagnosed when anatomical changes of the trigeminal nerve root, such as atrophy or displacement, attributable to neurovascular compression are identified. Secondary trigeminal neuralgia occurs in association with underlying disorders, including cerebellopontine angle tumors, arteriovenous malformations, and multiple sclerosis. In the presence of trigeminal neuralgia, when a neurovascular contact is observed but there is no radiological or intraoperative evidence of morphological changes in the nerve root, such as atrophy or displacement, the condition is classified as idiopathic trigeminal neuralgia. However, the etiology and optimal therapeutic strategies for idiopathic trigeminal neuralgia remain poorly understood. Traditionally, nerve combing, also referred to as internal neurolysis, has been described as an additional surgical option for patients with refractory trigeminal neuralgia (2). This procedure involves longitudinally splitting the trigeminal nerve root into multiple fascicles from the root entry zone (REZ) to Meckel's cave. In this report, we present two cases of idiopathic trigeminal neuralgia successfully treated with nerve combing and discuss the potential role of this procedure in the management of idiopathic trigeminal neuralgia.

## Case Report

**Figure 1:**
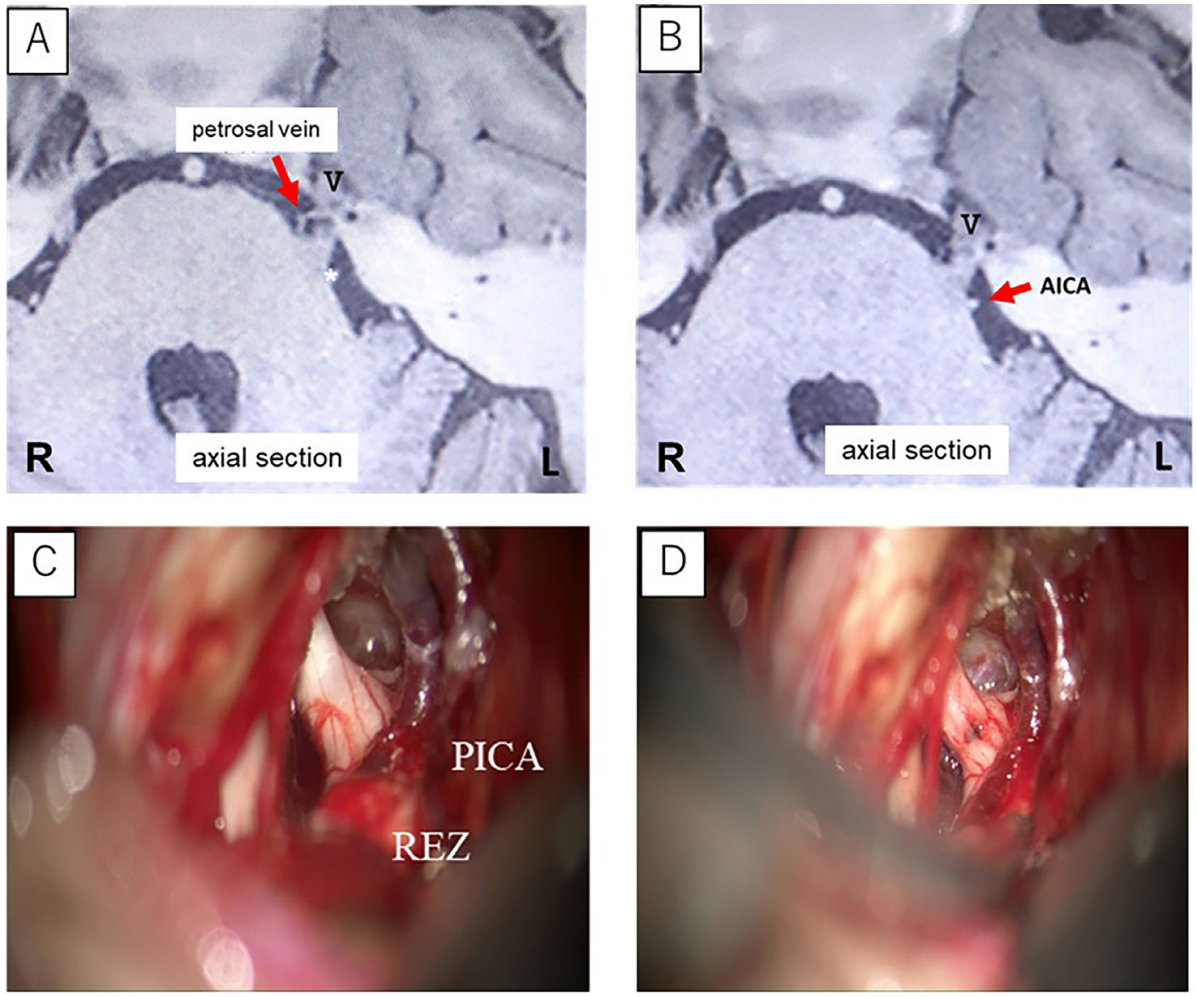
Case 1: (A) Preoperative cranial magnetic resonance imaging (MRI) shows the petrosal vein is not in contact with the trigeminal nerve root. (B) The anterior inferior cerebellar artery (AICA) does not contact the trigeminal nerve root. (C) Intraoperative findings reveal the posterior inferior cerebellar artery (PICA); however, no arterial or venous structures are in contact with the trigeminal nerve root. (D) The trigeminal nerve after nerve combing.

Case 1 A 44-year-old woman presented with a history of pain involving the left nasal ala, cheek, and upper lip that had persisted for more than one year. She initially consulted a local dental clinic, where endodontic treatment was performed on multiple teeth; however, her symptoms did not improve. The pain severity was rated as 9-10 on the Numerical Rating Scale (NRS) and was characterized by paroxysmal, electric shock-like attacks lasting several tens of seconds, occurring both at rest and during speaking or face washing. Because her symptoms were refractory to dental treatment, she was referred to our department for further evaluation and management. Her past medical history was unremarkable. (Fig. 1).

1

**Figure 2:**
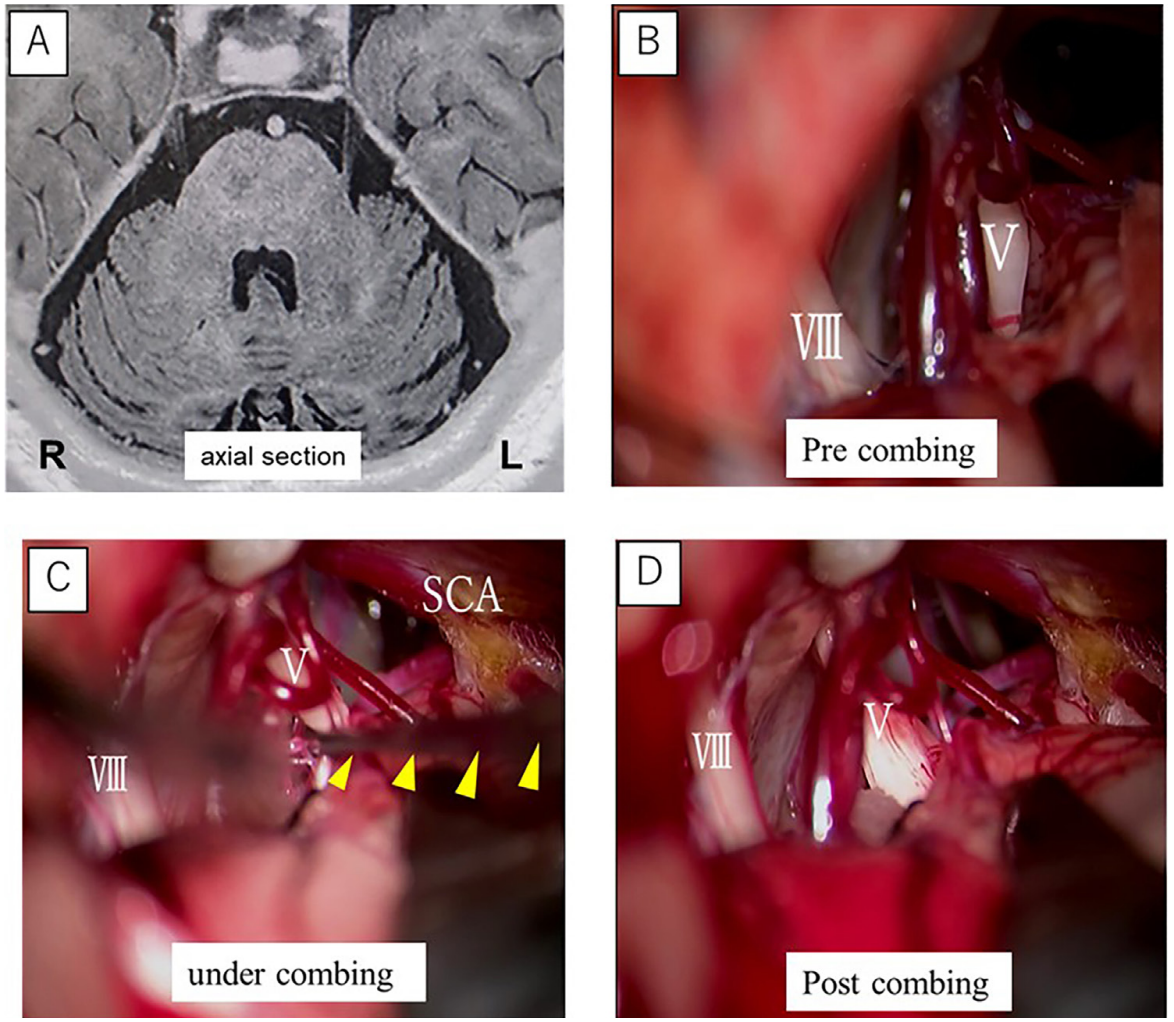
Case 2: (A) Preoperative cranial magnetic resonance imaging (MRI) shows no evidence of neurovascular compression of the trigeminal nerve root. (B) Anatomical appearance of the trigeminal nerve root before nerve combing. (C) Intraoperative view during nerve combing; the yellow arrow indicates the surgical instrument longitudinally incising the trigeminal nerve. (D) The trigeminal nerve after nerve combing.PICA: Posterior Inferior Cerebellar Artery. AICA: Anterior Inferior Cerebellar Artery. SCA: Superior Cerebellar Artery.

Examination findings During the clinical examination, trigger zones were identified on the left upper lip and nasal ala. Based on the characteristic pain features and the presence of trigger zones, a diagnosis of trigeminal neuralgia was made. Magnetic resonance imaging (MRI) demonstrated contact between the left trigeminal nerve and the anterior inferior cerebellar artery (AICA). A petrosal vein was observed in close proximity to the nerve. However, there was no evidence of neurovascular compression. Following a diagnostic trial of carbamazepine (CBZ), the frequency of pain attacks decreased, and pain intensity was markedly reduced to NRS 1-2, indicating a pronounced analgesic effect. However, at a dose of 100 mg per day, the patient developed a generalized drug eruption involving the extremities and trunk, necessitating hospitalization in the dermatology department. Because adequate sustained pain control with CBZ was not feasible, the patient was referred to the Department of Neurosurgery. She subsequently underwent posterior fossa craniotomy with a microvascular decompression protocol, combined with nerve combing. Intraoperatively, no vessel was identified as clearly causing neurovascular compression; however, axial torsion of the trigeminal nerve root caused by thickened arachnoid adhesions was observed. The arachnoid membranes surrounding the nerve and adjacent vessels were carefully dissected to allow mobilization of all contacting vessels. The epineurium of the trigeminal nerve was then incised using an arachnoid knife, and nerve combing was performed at approximately three sites. Postoperatively, the patient's pain resolved completely. Mild hypoesthesia of the left facial skin persisted for approximately two months but subsequently resolved with full sensory recovery. Case 2 A 73-year-old man presented with a one-year history of pain localized to the left buccal gingiva. The pain was characterized as electric shock-like, with an intensity of NRS 7-8, lasting for several seconds, and was triggered by mastication, tooth brushing, and shaving. Despite undergoing pulpectomy of the left maxillary molar region at a dental clinic, his symptoms did not improve. Subsequently, he developed paroxysmal, electric shock-like pain in the skin corresponding to the left nasal ala and sought evaluation at a local internal medical clinic. Computed tomography revealed no abnormal findings. He was prescribed loxoprofen and pregabalin; however, his pain persisted, and he was referred to our department for further assessment. He had no significant past medical history, (Fig. 2).

2

Examination findings During the clinical examination, trigger zones were identified on the left upper lip and nasal ala. Based on the characteristic pain features and the presence of trigger zones, a diagnosis of trigeminal neuralgia was established. A diagnostic trial of CBZ at a dose of 100 mg resulted in pain relief, with a reduction in pain intensity to NRS 0. Although the CBZ dose was gradually increased from 200 mg to 400 mg, adequate pain control became increasingly difficult to achieve, prompting referral to the Department of Neurosurgery. Magnetic resonance imaging demonstrated contact between the left trigeminal nerve and the superior cerebellar artery (SCA), without evidence of definite neurovascular compression. The patient subsequently underwent posterior fossa craniotomy with a microvascular decompression protocol combined with nerve combing. Preoperatively, auditory brainstem response, motor evoked potential, somatosensory evoked potential, and nerve integrity monitoring were established with the patient in the right lateral decubitus position. In addition, compound nerve action potential (CNAP) monitoring was employed. Intraoperatively, two branches of the SCA were identified coursing dorsally in close proximity to the trigeminal nerve, although no clear compressive pathology was observed. The arachnoid membranes surrounding the nerve were dissected, and the nerve sheath was incised to perform nerve combing, which ultimately resulted in correction of the axial distortion of the trigeminal nerve. Prior to microvascular decompression, abnormal waveforms were observed with CNAP monitoring; as the surrounding arachnoid adhesions were released, the amplitude gradually increased, whereas subsequent nerve combing was associated with a reduction in the recorded potentials. Postoperatively, the patient's pain resolved. Transient hypoesthesia of the left facial skin developed, but improved within one month.

## Discussion

Based on etiology, trigeminal neuralgia is classified into secondary trigeminal neuralgia associated with major neurological disorders, classical trigeminal neuralgia related to neurovascular compression causing anatomical changes of the trigeminal nerve root-including displacement, atrophy, and large-fiber demyelination-and idiopathic trigeminal neuralgia of unknown cause (3 , 4). According to the ICOP, contact between a blood vessel and the trigeminal nerve and/or its root is a frequent neuroimaging finding even in healthy individuals (1). In the presence of trigeminal neuralgia, when such neurovascular contact is observed without radiological or intraoperative evidence of morphological changes in the trigeminal nerve root, such as atrophy or displacement, the diagnostic criteria for classical trigeminal neuralgia (ICOP 4.1.1.1) are not fulfilled, and the condition should therefore be classified as idiopathic trigeminal neuralgia. Further elucidating the clinical and neurophysiological differences between classical and idiopathic trigeminal neuralgia may contribute to a better understanding of their shared yet distinct pathophysiological mechanisms. This may be particularly valuable in cases in which MRI findings are inconclusive, as differentiation may allow more precise clinical management (4). Only a limited number of studies have investigated nerve combing as a treatment for trigeminal neuralgia. The application of nerve combing for trigeminal neuralgia without evidence of neurovascular conflict was first reported by Li et al. in 1995 (5). Although the precise mechanism by which nerve combing achieves pain relief remains unclear, it has been suggested that the procedure induces axonal injury, followed by irreversible degeneration of a portion of the axonal tissue and myelin sheath over time. In addition, nerve combing may disrupt interneuronal connections among trigeminal nerve fibers and interfere with communication between different trigeminal divisions prior to their entry into the brainstem. In both of the present cases, intraoperative findings revealed thickened arachnoid membranes and adhesions between the trigeminal nerve root and surrounding structures, resulting in axial torsion of the trigeminal nerve root. In Case 2, abnormal waveforms were observed on CNAP monitoring prior to microvascular decompression. As the surrounding arachnoid membranes were progressively dissected, the CNAP amplitude increased, whereas subsequent nerve combing was associated with a reduction in the recorded potentials. These findings suggest that axial torsion of the trigeminal nerve root caused by thickened arachnoid membranes may promote neural hyperexcitability and contribute to pain generation. Dissection of the arachnoid adhesions may gradually restore a more linear and relaxed configuration of the nerve, while additional nerve combing may further alleviate paroxysmal pain by disrupting pathological interconnections among trigeminal nerve fibers and aberrant signal transmission between trigeminal divisions (2). Li et al. reported on internal neurolysis performed in a subgroup of 36 patients in whom no neurovascular compression was identified intraoperatively, achieving a medium- to long-term pain relief rate of 91.7% (6 , 7). In the present study, Case 1 has been followed for 22 months postoperatively and Case 2 for 13 months, and both patients continue to show excellent outcomes with a NRS score of 0. Postoperative sensory disturbances following IN have been reported in a series of 10 patients (8). Transient sensory deficits involving all or part of the trigeminal sensory branches-affecting touch, warm, and cold sensation-gradually improved in 9 patients. The mean duration of postoperative sensory disturbance was 5.35 months, with recovery occurring within several months in most cases. Permanent sensory deficits were observed in one patient, persisting for 36 months. In the present cases, sensory disturbances persisted for 2 months in Case 1 and for 1 month in Case 2, with complete resolution in both. Although the duration of postoperative sensory impairment differed from previous reports, this difference may be attributable to several surgical factors, including the optimal number of fascicles to be dissected, the extent of neurolysis, whether the minor portion of the trigeminal nerve is included, and the intrinsic consistency of the nerve itself (9). In the present cases, a diagnostic trial of CBZ demonstrated high clinical utility in the diagnosis of idiopathic trigeminal neuralgia. In Case 1, the patient presented with severe paroxysmal pain with an NRS score of 9-10 at the initial visit, which markedly improved to NRS 1-2 following diagnostic administration. In Case 2, prior treatment with loxoprofen and pregabalin at another institution was ineffective; however, diagnostic CBZ administration resulted in a clear reduction in pain intensity from NRS 7-8 to complete pain relief (NRS 0). Even in the absence of neurovascular compression on MRI, such a characteristic pharmacological response is highly suggestive of trigeminal neuralgia. This has diagnostic value in the dental office, and prompt diagnosis also avoids unnecessary and irreversible dental procedures. Diagnostic administration of CBZ should be actively considered in patients suspected of having idiopathic trigeminal neuralgia.

## Conclusions

These cases demonstrate that axial torsion of the trigeminal nerve root caused by thickened arachnoid adhesions may represent an underlying etiological factor in idiopathic trigeminal neuralgia, and that careful dissection of perineural arachnoid adhesions to restore a linear configuration of the nerve root should be considered as part of the surgical strategy. Furthermore, nerve combing appears to be an effective therapeutic option for eliminating postoperative paroxysmal pain attacks in patients with idiopathic trigeminal neuralgia. In dental clinical practice, even in the absence of neurovascular compression on MRI, a diagnostic trial of CBZ represents a highly valuable adjunctive tool for the diagnosis of idiopathic trigeminal neuralgia and contributes meaningfully to appropriate diagnostic accuracy and subsequent treatment decision-making.

## Data Availability

The datasets used and/or analyzed during the current study are available from the corresponding author.
